# Fertility-sparing uterine lesion resection for young women with gestational trophoblastic neoplasias: single institution experience

**DOI:** 10.18632/oncotarget.14727

**Published:** 2017-01-18

**Authors:** Xiaoyu Wang, Junjun Yang, Jie Li, Jun Zhao, Tong Ren, Fengzhi Feng, Xirun Wan, Yang Xiang

**Affiliations:** ^1^ Department of Obstetrics and Gynecology, Peking Union Medical College Hospital, Chinese Academy of Medical Sciences and Peking Union Medical College, Beijing, PR China

**Keywords:** gestational trophoblastic neoplasias, fertility-sparing surgery, uterine lesion resection, prognostic factor, pregnant outcomes

## Abstract

**Purpose:**

To evaluate the oncological safety and pregnant outcomes of fertility-sparing uterine lesion resection in treating gestational trophoblastic neoplasias.

**Results:**

After the treatment of surgery and chemotherapy, all the patients achieved complete remission. With a median follow-up time of 44 months (range, 6-188), 3 patients (3.85%) relapsed within 3-26 months. Multivariate analysis showed that tumor size was the independent risk factor of recurrence and the cutoff value was 4.2cm. Among 37 patients who attempted to conceive, 31 achieved clinical pregnancy. The rate of pregnancy and live birth were 83.8% and 77.4%. Uterine rupture did not occurred no matter in cesarean section or vaginal delivery. No congenital abnormalities were reported among the live births.

**Methods:**

From January 1995 to December 2014, 78 patients with gestational trophoblastic neoplasias who underwent fertility-sparing uterine lesion resection at Peking Union Medical College Hospital were reviewed. The complete remission rate, fertility rate, pregnant outcomes and risk factors of recurrence were analyzed.

**Conclusions:**

Fertility-sparing uterine lesion resection might be considered as a safe and reasonable alternative for high-selected young women to remove uterine lesion in the treatment of gestational trophoblastic neoplasias.

## INTRODUCTION

Gestational trophoblastic neoplasias (GTNs) used to describe a spectrum of diseases derived from trophoblasts and often occurred in young women of child-bearing age. GTNs are highly responsive to chemotherapy especially in choriocarcinomas even with distant metastasis. Although surgery was considered as a less important approach in the management of GTNs, selected surgical procedures may be necessary for removing chemo-resistant or persistent lesions in uterus and metastatic sites, and for curing severe complications [1]. Hysterectomy is recommended when lesion is localized in uterus, but loss of fertility is the major concern for young women. Previous studies have indicated that the fertility-sparing uterine lesion resection for GTNs is well tolerant, which provides an alternative to eliminate lesion and preserve the fertility function [2, 3]. However, uterine lesion resection may increase the chance of tumor spread and recurrence so the oncological safety must be balanced. There are several cases of successful pregnancy and delivery after this surgery; in the same time, the risk of uterine rupture is also controversial [4]. The purposes of our study were to analyze the clinical characteristics and oncological safety, as well as the prognosis and reproductive outcomes of patients with GTNs undergoing fertility-sparing uterine lesion resection in Peking Union Medical Collage Hospital (PUMCH) within the past 20 years.

## RESULTS

### Patient characteristics

The demographic characteristics of enrolled 78 patients before treatment in our hospital were displayed in Table 1. With the mean age of 26.5 years (range, 16-38 years), 63 patients (80.8%) were nulliparae. The mean serum β-hCG level before treatment was 154873.2 (range, 15.5-3508811) IU/L. 43 patients (55.1%) were low-risk according to the International Federation of Gynecology and Obstetrics (FIGO) prognostic scoring system. 42 patients were diagnosed with stage I, 1 with stage II, 31 with stage III, and 4 with stage IV. The percentage of patients with metastases was 46.2% (36/78), which included 34 cases with lung metastases, 2 with liver metastases, 1 with vaginal metastasis, 1 with brain metastasis and 1 with pelvic wall metastasis. 8 patients had GTN history before and 10 patients were transferred to our hospital because resistant to multi-drug chemotherapy in local hospitals.

**Table 1:** Clinical features of the 78 patient before treatments in our hospital

Characteristics	*NO*. (Ratio) or Mean (Range)
Age	26.5 (16-38)
Gravidity	2.0 (1-6)
Parity time	
0	63 (80.8%)
1	12 (15.4%)
2	3 (3.8%)
Pretreatment β-HCG (IU/L)	154873.2 (15.5-3508811)
FIGO score	
≤6	43 (55.1%)
≥7	35 (44.9%)
FIGO stage	
I	42 (53.9%)
II	1 (1.3%)
III	31 (39.7%)
IV	4 (5.1%)
Metastasis	
Lung	34 (43.6%)
Liver	2 (2.6%)
Brain	1 (1.3%)
Vagina	1 (1.3%)
Pelvic wall	1 (1.3%)
Previous GTN history	
NO	70 (89.7%)
YES	8 (10.3%)
Resistant to multi-drug chemotherapy	
NO	68 (87.2%)
YES	10 (12.8%)

### Treatments

The median course of chemotherapy before surgery was 3.5 (range, 0-12). The mean preoperative serum β-hCG level was 4778.3 (range, 0-213857) IU/L. The indications for surgery were persistent lesion, chemo-resistant lesion, unclear diagnosis and suspected uterine rupture, in 51 patients (65.4%), 13 patients (16.7 %), 9 patients (11.5%) and 5 patients (6.4%), respectively. The mean preoperative serum β-hCG level in above was 0.7 (range, 0-4.4) IU/L, 123.8 (range, 5.8-1294) IU/L, 494.9 (range, 15.8-3071.3) IU/L, and 73321.2 (range, 14160-213857) IU/L, respectively. The mean size of lesion in the uterus was 3.7 (range, 3.0-8.1) cm. The median postoperative chemotherapy course was 3 (range, 0-6). Combined medical history, previous and present histological and immunohistochemical results, the 78 patients were diagnosed as the following: 26 invasive moles, 40 choriocarcinomas and 12 placental site trophoblastic tumors (PSTTs). These included 3 invasive moles and 2 choriocarcinomas in 5 patients with uterine rupture, and 8 PSTTs and 1 choriocarcinoma in 9 patients with unclear diagnosis before surgery. Detailed treatment information was showed in Table 2.

**Table 2:** Clinical features of the 78 patient within treatments

Characteristics	*NO*. (Ratio) or Mean (Range)
Surgical indications	
Persistent lesion	51 (65.4%)
Chemo-resistant lesion	13 (16.7%)
Unclear diagnosis	9 (11.5%)
Suspected uterine rupture	5 (6.4%)
Median preoperative chemotherapy course	3.5 (0-12)
Preoperative serum β-HCG (IU/L)	4778.3 (0-213857)
Persistent lesions	0.7 (0-4.4)
Chemo-resistant lesions	123.8 (5.8-1294)
Unclear diagnosis	494.9 (15.8-3071.3)
Suspected uterine rupture	73321.2 (14160-213857)
Uterine lesion size in the surgery (cm)	3.7 (3.0-8.1)
Postoperative serum β-HCG (IU/L)	2372.2 (0-126321)
Persistent lesions	0.3 (0-2.9)
Chemo-resistant lesions	47.5 (0.2-567)
Unclear diagnosis	76.8 (2.3-456)
Suspected uterine rupture	36741.2 (5100-126321)
Median postoperative chemotherapy course	3 (0-6)

### Follow-ups

After the completion of treatment, all patients achieved complete remission (CR). The median follow-up time was 44 months (range, 6-188). Three of 78 patients relapsed after CR within 3-26 months, one of which had three times of recurrence; the recurrent rate was 3.85%. Table 3a and Table 3b showed the clinical characteristics of 3 patients who relapsed after the completion of treatment. They received chemotherapy with or without surgery when relapsed and one of them died due to tumor progression.

In the follow-up time, 41 patients selected continuous contraception because of young age, having child before, or for fear of recurrence. In the other 37 patients, 31 were successful pregnant (83.8%). There were 22 term deliveries (71.0%), 2 preterm deliveries, 1 uterine scar pregnancy, 1 cornual pregnancy, 1 spontaneous abortion, 1 mid-term induced abortion due to fetus cleft lip, 1 repeated hydatidiform mole, and 2 on-going pregnancies in their first pregnancies. No congenital abnormalities were reported among the live births. Among the 24 term and preterm deliveries, the cesarean section accounted for 83.3% (20/24) and uterine rupture not occurred no matter in cesarean section or vaginal delivery.

**Table 3a:** Clinical characteristics of the 3 patients relapsed after treatments

No	Age	Gravidity	Parity	GTN type	FIGO stage	FIGO score	Metastasis	Pretreatment β-HCG	Previous GTN history	Resistant to multi-drug	Surgical indication	Preoperative chemotherapy (regimen*n.)
1	23	1	0	CC	IV	11	Lung, liver	981100	NO	YES	Chemo-resistant lesion	Other hospital*2 (5FU+Act-D*2) FAEV*2 EMA/CO*4
2	38	5	0	CC	I	11	NO	2383	YES	NO	Chemo-resistant lesion	FAEV*4 EMA/CO*3
3	27	4	0	CC	I	8	NO	60000	NO	YES	Persistent lesion	Other hospital*5 (PEB*3, EMA/CO*2) EMA/CO*3

Abbreviations: CC, choriocarcinoma; 5FU, 5fluorouracil; Act-D,dactinomycin; EMA/CO, etoposide+methotrexate+dactinomycin/cyclophosphamide+vincristine; FAEV, floxuridine+dactinomycin+vincristine+etoposide; PEB, cisplatin+etoposide+bleomycin.

**Table 3b:** Clinical characteristics of the 3 patients relapsed after treatment

No	Preoperative β-HCG (IU/L)	Surgical mode	Tumor size (cm)	Postoperative β-HCG (IU/L)	Postoperative chemotherapy (regimen*n.)	Time to recurrence (months)	Recurrent site	Recurrent treatment	Status (months)
1	120	Laparotomy	3.3	21	EMA/CO*5	6	Uterus, brain, lung	Chemotherapy	DOD (16)
2	10.5	Laparotomy	5.6	0.6	EMA/CO*3	3	Uterus, lung	Chemotherapy, pulmonary lobectomy	NED (7)
3	1.6	Laparotomy	5.2	0	EMA/CO*3	3, 12, 26	Uterus, lung	Chemotherapy, hysterectomy	NED (45)

Abbreviations: DOD, dead of disease; NED, no evidence of disease.

### Statistical analysis

In univariate analysis, resistant to multi-drug chemotherapy, FIGO score and tumor size were significantly associated with recurrence, which were shown in Table 4 and Table 5. A multivariate analysis of aforementioned factors identified the tumor size (OR = 6.184, 95% CI: 1.090-35.071, *P* = 0.040) was an independent risk factor for recurrence. Different tumor sizes were used to calculate the sensitivity, specificity and Youden's index, and these results revealed that the maximal Youden's value correlated with a tumor diameter of 4.2cm (Sensitivity 1.0, Specificity 0.8, Youden's index 0.8). It means that patients with tumor diameter of 4.2cm or above have more possibilities of recurrence after uterine lesion resection surgery.

**Table 4:** Results of the univariate analysis for recurrence (qualitative indicators)

	Totality (*n*,%)	No recurrence (*n*,%)	Recurrrence (*n*,%)	*P* value
Total number	78	75 (96.2%)	3 (3.8%)	
Resistant to multi-drug chemotherapy				
NO	68 (87.2%)	67 (98.5%)	1 (1.5%)	0.04
YES	10 (12.8%)	8 (80%)	2 (20%)	
Previous GTN history				
NO	70 (89.7%)	68 (97.1%)	2 (2.9%)	0.28
YES	8 (10.3%)	7 (87.5%)	1 (12.5%)	
Surgical indications				
Persistent lesion	51	50 (98.1%)	1 (1.9%)	0.10
Chemo-resistant lesion	13	11 (84.6%)	2 (15.4%)	
Unclear diagnosis	9	9 (100%)	0	N/A
Suspected uterine rupture	5	5 (100%)	0	
FIGO stage				
I	42	40 (92.9%)	2 (7.1%)	N/A
II	1	1 (100%)	0	
III	31	31 (100%)	0	
IV	4	3 (80%)	1 (20%)	
Surgical mode				
Laparotomy	56 (71.8%)	53 (94.6%)	3 (5.4%)	0.56
Laparoscopy	22 (28.2%)	22 (100%)	0	

Abbreviations: N/A, not applicable.

**Table 5:** Results of the univariate analysis for recurrence (quantitative indicators)

	Category	Case	Mean±SD	Median	InterQuartile range	Minimum–maximum	Z	*P*
Age	1	75	26.43±4.71	26.00	7.00	16.00-42.00	−0.639	0.553
	2	3	29.33±7.68	27.00	15.00	23.00-38.00		
FIGO score	1	75	5.79±2.815	6.00	4.00	1.00-12.00	−2.313	0.015
	2	3	10±1.732	11.00	3.00	8.00-11.00		
Tumor size	1	75	3.62±0.74	3.30	1.00	3.00-5.60	−2.556	0.005
	2	3	5.87±1.99	5.20	3.80	4.80-8.10		

Abbreviations:1, group of not recurrence after treatments; 2, group of recurrence after treatments.

## DISCUSSION

With the development of sensitive assays of β-hCG, the effective chemotherapy regimens and the recognition of risk factors, the cure rate of GTNs could almost reach 100% in low-risk patients even with metastasis and 94% in high-risk patients [5]. However, it is important to remove the chemo-resistant or persistent lesions through surgery in order to cure disease and reduce the chance of relapse. With an incidence ranging from 5.2% to 23.4%, many centers had reported their experience of hysterectomy in the management of uterine lesion [1, 6–8]. Fertility-sparing uterine lesion resection, which combined conservative myometrium resection with uterine reconstruction, can be considered in highly-selected patients who desire for preserving future fertility. However, compared to the vast reports about hysterectomy in management of GTNs, there was a paucity of publication describing fertility-sparing surgery, especially regarding rare types. Therefore, retrospective analysis of prognosis and pregnancy outcomes may contribute to establish better therapeutic strategies for young patients with GTNs, particularly when it comes to the fertility-sparing surgery.

Once definitely diagnosed for GTN, chemotherapy is the primary approach, both in low-risk and high-risk patients. However, rapid growth and massive hemorrhage of tumors may result in a surgical emergency. Uterine perforation due to myometrium invasion is one of these emergent conditions. Uterine arterial embolization (UAE) is a safe and effective treatment for terminating vaginal excessive bleeding. The purposes of fertility-sparing emergent surgery were to stop bleeding, establish diagnosis and remove lesions as possible. Patients should be well informed about surgical risk and intraoperative frozen pathology was necessary. No severe intraoperative and postoperative complication was occurred in our study. The pathologic results were 3 invasive moles and 2 choriocarcinomas. All the 5 patients received powerful chemotherapy after surgery and achieved complete remission. In the follow-up times, none of them relapsed and 2 had successful pregnancies (the other 3 patients were in contraception). Therefore, as for uterine rupture with invasive mole or choriocarcinoma, uterine lesion resection combined with subsequent chemotherapy may be a feasible and safe therapeutic strategy.

Sometimes it is difficult to distinguish incomplete abortion, cornual pregnancy, interstitial tubal pregnancy, and cesarean scar pregnancy from GTNs, especially with a low serum β-hCG level. The symptoms of amenorrhea, vaginal bleeding, pelvic pain and the imaging features of uterine mass with abundant blood flow are similar to some extent. Timely diagnosis could avoid progression of disease and shorten treatment time. Curettage may provide histologic evidence but sometimes it is difficult to obtain tissues. Suzuki [9] reported hysteroscopy could not only confirm complete evacuation of hydatidiform mole but also contribute to the diagnosis and management of invasive mole and choriocarcinoma. As to lesions away from uterine cavity, laparotomic or laparoscopic exploration may provide a better strategy in the diagnosis and differentiation, and treatment of GTNs. In our series, chemotherapy was administered after pathological examination and CR was obtained in all 9 patients.

PSTT is a rare type of gestational trophoblastic neoplasias, derived from intermediate cytotrophoblasts, accounted for 0.5-3% of all type of GTNs according to population-based study [10, 11]. Surgery plays an important role in the treatment of PSTT because it was not sensitive to chemotherapy. Hysterectomy is widely accepted as the primary treatment for PSTT confined to the uterus. Shen [12] and Zhao [10] had summarized a series of PSTT cases which received fertility-sparing therapy, included diagnostic curettage, resection of lesion by hysteroscopy, and laparoscopic or laparotomic uterine lesion resection. The age older than 35 years may be a risk factor for prognosis [12]. Although there were not enough literatures about uterine lesion resection with PSTT [10, 12–15], the prognosis and fertility outcomes were encouraging. However, as to patients with large tumor size, multiple metastases or finished childbearing, uterine lesion resection was not recommended. In our series, there were 12 patients receiving this surgery, including 9 with stage I and 3 with stage III. All the 12 patients were younger than 35 years, untreated cases and had no GTN history. 1 patient only received surgery and the other 11 accepted postoperative chemotherapies. In the follow-up time, none of the 12 patients relapsed and 5 of them had term deliveries.

Our data showed that the recurrent rate of patients receiving uterine tumor resection was 3.85%, which was similar to all GTNs reported in other literatures (4%-8%) [16, 17]. Univariate analysis showed that resistant to multi-drug chemotherapy, FIGO score and tumor size were significantly associated with recurrence. And the result of multivariate analysis implied the tumor size was the independent risk factor. Higher FIGO score and chemo-resistance were the risk factors reported in many literatures [8, 18, 19]. Even though surgery could improve the prognosis especially for patients with chemo-resistant and recurrent GTNs [20], uterine lesion resection need to be paid more attention. Due to lack of big sample about lesion resection surgery in other centers, the suitable tumor size for surgery is not definite. Large lesion especially with unclear border may increase the risk of tumor residues. Although the result of our study showed that the tumor size was the independent risk factor of recurrence and the cutoff value was 4.2cm, it should be noted that, the 95% of confidence interval in multivariate analysis was relative broad due to only 3 cases relapsed in our series. Larger sample was needed to confirm this finding.

Spontaneous abortion and adverse maternal outcomes tended to occur in patients conceived within 6 months after chemotherapy completion [21], and untimely pregnancy after surgery may increase the risk of uterine rupture, so patients are advised for contraception at last 12 months after completion of treatment in our center. Some of patients may express worries about future pregnancies, particularly the recurrence of GTNs, adverse pregnancy outcomes and fetal malformations [22], which are the same concerns of doctors before clinical decisions. With a theoretical risk of ovarian dysfunction, resumption of normal menstruation appears in almost 95% of women treated with chemotherapy for GTNs [23]. In our series, except 2 patients were amenorrhea after chemotherapy, the other 76 patients (97.4%) resumed menstruation within 2 to 6 months. A review article summing up the data from 7 international medical centers showed that the rate of term live delivery in women treated with chemotherapy for GTNs was higher than 70% without increased risk of congenital abnormalities [26]. Woolas [27] summarized the rate of pregnancy was 83% in patients with GTNs after methotrexate (MTX) or EMA/CO (etoposide, MTX, dactinomycin/cyclophosphamide, vincristine) chemotherapy. Besides the chemotherapy, uterine surgery also has influence on fertility outcomes, which may have potential risk of scar pregnancy, abortion and uterine rupture. Behtash [3] reported 2 cases with choriocarcinomas were successful pregnant after localized resection of perforated uterus. In our study, 37 patients attempted pregnancy and 31 succeeded (83.8%). In the 31 gestations, the rate of live birth and term delivery was 77.4% and 71.0% respectively, while the rate of premature deliveries, miscarriage, repeated mole, scar pregnancy and fetal abnormalities were 6.5%, 3.2%, 3.2%, 3.2% and 3.2%, respectively. Braga [21] reported pregnant outcomes of 252 patients with GTNs receiving chemotherapy alone, and the rate of term deliveries, preterm deliveries were 68.2% and 4.2%, which were similar to our data; while the rate of spontaneous abortion was 16.7%, which may related to some patients conceiving within 6 months after chemotherapy. Intraoperative suture carefully and layer by layer may decrease the risk of uterine rupture in future pregnancy. Even that elective cesarean section was recognized a better way to ensure maternal and perinatal safety, 4 term infants were delivered vaginally without uterine rupture in our study.

In conclusion, fertility-sparing uterine lesion resection might be considered as a safe and reasonable alternative for highly-selected young women with GTNs including PSTTs; all the patients achieved CR after surgery and chemotherapy in our series. Even in the emergency with uterine perforation, and under the situation of unclear diagnosis, localized resection combined subsequent chemotherapy also could be a safe method to cure disease and preserve fertility. Although our sample was limited, the recurrent rate was only 3.85% and similar to all GTNs, but more attention should be placed on these with larger tumor size. The rate of fertility and pregnant outcomes were satisfied as chemotherapy alone. Neither uterine rupture nor neonatal abnormality was found in our data. However, long term follow-up and additional samples would be necessary to confirm our findings.

## METERIALS AND METHODS

The retrospective study collected 78 patients with GTNs who underwent fertility-sparing uterine lesion resection from January 1995 to December 2014 in PUMCH. Information was reviewed by collecting medical files and making telephone interviews. This study was approved by the Institutional Review Board of PUMCH.

### Preoperative treatments

Before treatment, all patients received disease evaluations consisting of complete medical history reviews, blood sample tests of serum β-hCG levels, common blood counts, liver and renal function measures, and distant metastatic organs assessment. Pelvic examinations including ultrasound, CT or MRI were necessary to evaluate uterine lesion. Patients were scored and staged on the basis of the FIGO scoring/staging system 2000. Among the 78 patients, 67 accepted chemotherapy as primary treatment. Different chemotherapy agents were chosen due to tumor diagnosis and FIGO score. As to low-risk patients, single agent chemotherapy such as dactinomycin or MTX was administered. Other patients received multi-drug chemotherapy, regimens containing FAEV (floxuridine, dactinomycin, etoposide, vincristine), FAV (floxuridine, dactinomycin, vincristine) [28, 29], EMA/CO, EMA/EP (etoposide, MTX, dactinomycin/etoposide, cisplatin). The toxicity of chemotherapy and serum β-hCG were examined weekly and pelvic ultrasound was evaluated every 2-3 courses. Chemo-resistance was diagnosed when serum β-hCG not decline logarithmically, remained or rose after 2 consecutive chemotherapy courses.

### Perioperative treatments

The indications of uterine lesion resection were suspected uterine rupture, unclear diagnosis, and diameter of chemo-resistant (serum β-hCG > 5IU/L) or persistent (serum β-hCG < 5IU/L) lesions larger than 3cm. Diffuse and more than one lesion in uterus were the contradiction of surgery. Surgical risk was fully informed to patients and their families before operation. Patients who were suspected uterine rupture received UAE before emergent surgery. A total of twenty-two patients (28.2%) received laparoscopic uterine lesion resection. During the surgery, pelvic and abdominal organs were inspected carefully in order to reconfirm the lesion's location and size. The operative range included the whole lesion and surrounding tissue about 0.5-1cm as possible. Uterus was reconstructed after myometrium and seromuscular layer sutured separately. MTX or 5-floxuridine was multiple injected around uterine incision. The intraoperative frozen pathology was examined especially in the emergency of uterine perforation and unclear diagnosis before surgery. Chemotherapy was administered in definitely diagnostic patients during perioperative period.

### Postoperative treatments

Except one patient only received surgery, other 77 patients underwent subsequent and consolidation chemotherapy after surgery. The regimens were the same as preoperative. After the completion of treatment, all the patients underwent regularly and long-term follow-up for serum β-hCG. Complete remission was obtained when serum β-hCG decreased to normalization at least 4 consecutive weeks. While relapse was diagnosed as serum β-hCG increased 3 months after complete remission.

### Statistical analysis

All statistical analyses were performed with the Statistical Package for the Social Science, version 16.0 (SPSS 16.0, Chicago, IL, USA). The frequency distributions were compared by Fisher Exact Test. Wilcoxon Rank Sum Test was applied for quantitative parameters. Logistic regression method was used for the multivariate analysis. P value less than 0.05 was considered to be statistically significant.
